# Direct RNA sequencing reveals multilayered epitranscriptomic remodeling in macrophages upon Mtb infection

**DOI:** 10.3389/fcimb.2025.1689553

**Published:** 2025-11-11

**Authors:** Ren-Chao Zou, Kun Su, Dong Wei, Qiuhong Wang, Bo Tang, Tao Wang, Lianming Wang, Peng Chen, Tao Wu, Xiawei Yang

**Affiliations:** Department of Hepatobiliary Surgery, The Second Affiliated Hospital of Kunming Medical University, Kunming, Yunnan, China

**Keywords:** direct RNA sequencing, infection, m6a, tuberculosis - epidemiology, macrophages

## Abstract

Understanding the host transcriptional and epitranscriptomic response to *Mycobacterium tuberculosis* (Mtb) infection is vital for decoding mechanisms of immune evasion and persistence. Here, we employed Oxford Nanopore Technologies (ONT)-based direct RNA sequencing (DRS) on human THP-1 macrophages infected with Mtb. This third-generation sequencing approach enables full-length transcript analysis and simultaneous detection of RNA modifications without reverse transcription or amplification. We uncovered extensive alternative splicing events, widespread shortening of poly(A) tails, and significant shifts in usage of proximal versus distal polyadenylation sites upon infection. Furthermore, we identified infection-induced changes in m6A, m5C, pseudouridine (Ψ), and inosine modifications across different genomic regions, with distinct motif preference and distribution shifts. Pathway enrichment analyses revealed these changes were associated with host responses to infection, inflammation, metabolism, and apoptosis. Our study provides a comprehensive epitranscriptomic landscape of macrophage responses to Mtb infection and highlights potential regulatory layers governing host-pathogen interaction.

## Introduction

Tuberculosis (TB), caused by *Mycobacterium tuberculosis*, remains a global health threat, responsible for over 10 million new infections and approximately 1.3 million deaths each year (World Health Organization, 2023; Hinman et al., 2021; Goletti et al., 2025). A hallmark of Mtb pathogenesis is its ability to survive and replicate within macrophages, effectively subverting innate immune defenses and altering host gene expression. Despite advances in transcriptomics using second-generation RNA sequencing (RNA-seq), limitations remain in resolving full-length transcripts, isoforms, and RNA modifications critical for post-transcriptional regulation (Parker et al., 2020; Hendra et al., 2022; Nguyen et al., 2022; Tavakoli et al., 2023). Previous studies have reported that the rapid advances in SGS and TGS are driving a paradigm shift in aquaculture research—from single-gene investigations to genome-wide approaches (Li et al., 2020).

Third-generation sequencing technologies, such as Oxford Nanopore and PacBio, allow direct sequencing of full-length RNA molecules with the added advantage of detecting native RNA modifications (Huber et al., 2023). Direct RNA sequencing (DRS), in particular, offers an unbiased view of mRNA landscapes without amplification, enabling accurate identification of transcript isoforms, polyadenylation (polyA) lengths, and base modifications including N6-methyladenosine (m6A), 5-methylcytosine (m5C), pseudouridine (Ψ), and inosine (I) (Liu et al., 2021; Zhang et al., 2022).

Epitranscriptomic regulation has emerged as a critical layer of control during immune responses to viral and bacterial infections (An et al., 2022; Cun et al., 2023; Li et al., 2024). For instance, m6A methylation has been implicated in modulating immune transcripts and cytokine production (Chu et al., 2024; Lin et al., 2024), while APA and polyA tail dynamics influence mRNA stability and translational efficiency (Sanqi et al., 2021). However, these RNA-level modifications remain poorly characterized in the context of TB.

We performed Direct RNA Sequencing (DRS) on THP-1 macrophages infected with *Mycobacterium tuberculosis* (MTB) using the third-generation sequencing platform from Oxford Nanopore Technologies (ONT). This approach sequences native RNA molecules directly—without reverse transcription or amplification—thus avoiding amplification bias and enabling simultaneous detection of RNA modifications, including methylation marks. DRS provides accurate profiling of alternative splicing events, fusion transcripts, and novel isoforms. In addition, it allows relatively precise estimation of poly(A) tail lengths, captures the true molecular characteristics of RNA, and enables reliable quantification of transcript expression levels. Nevertheless, DRS remains constrained by comparatively high error rates (5–12%, dominated by homopolymer indels), limited throughput per flow cell (≈5–10 million reads for the latest R10.4 chemistry), and elevated cost per base. Base-calling accuracy of modified residues is context-dependent and often requires orthogonal validation (e.g., miCLIP or mass spectrometry). Additionally, the requirement for microgram quantities of high-quality, full-length RNA can be prohibitive for low-input or degraded clinical samples, and the absence of a widely accepted community standard for modification calling hampers cross-study comparability. Continued improvements in nanopore chemistry, duplex sequencing, and AI-driven base-callers are expected to mitigate these limitations, positioning DRS as an increasingly powerful platform for quantitative, single-molecule transcriptomics.

In this study, we applied ONT-based DRS to investigate global transcriptomic and epitranscriptomic alterations in Mtb-infected THP-1 macrophages. We systematically analyzed changes in isoform usage, splicing, polyA regulation, and base modifications, and mapped them to functional pathways implicated in inflammation, metabolism, and host-pathogen interactions.

## Materials and methods

### Cell line and *Mycobacterium tuberculosis* H37Rv

Human THP-1 monocytes (ATCC TIB-202) were maintained in RPMI-1640 medium (Gibco) supplemented with 10% heat-inactivated fetal bovine serum (FBS; Gibco), 100 U/mL penicillin, and 100 μg/mL streptomycin at 37 °C in a humidified incubator with 5% CO_2_. Cells were routinely passaged every 2–3 days to maintain exponential growth. For macrophage differentiation, THP-1 monocytes were seeded at a density of 1 × 10^6^ cells/mL and treated with 100 nM phorbol 12-myristate 13-acetate (PMA; Sigma) for 48 hours, followed by a 24-hour rest period in PMA-free medium to allow recovery. Differentiated macrophages were then infected with Mycobacterium tuberculosis H37Rv at a multiplicity of infection (MOI) of 10 for 72 hours under BSL-3 containment.

Total RNA was extracted using TRIzol reagent (Invitrogen) according to the manufacturer’s instructions, and RNA integrity and concentration were assessed using an Agilent 2100 Bioanalyzer. Direct RNA sequencing libraries were prepared with the Oxford Nanopore SQK-RNA002 kit following the standard protocol and sequenced on the PromethION platform.

### SELECT assay

m^6^A stoichiometry was determined with the Epi-SELECT™ m^6^A Fraction Quantification kit (Epigenetek, R202106M-03). 2–3 µg RNA was split into two reaction sets:*Set N* (non-m^6^A reference) :9.8 µL RNA, 1.6 µL Up Probe-N (1 µM), 1.6 µL Down Probe-N (1 µM), 2 µL dNTP, 2 µL 10× Reaction Buffer (17 µL total). Reactions were temperature-cycled (90°C 1 min → 80°C 1 min → 70°C 1 min → 60°C 1 min → 50°C 1 min → 40°C 6 min; lid 105°C). After cycling, 0.3 µL SELECT™ DNA polymerase, 0.47 µL SELECT™ ligase and 2.23 µL ATP were added to each 17 µL reaction, adjusted to 20 µL, and incubated (40°C 20 min → 80°C 20 min → 4°C hold).

### qPCR quantification

One microliter of ligated product was used in a 10 µL qPCR reaction containing 5 µL ChamQ Universal SYBR qPCR Master Mix (Vazyme, Q711), 0.2 µL each Select primer (10 µM) and 3.6 µL nuclease-free water. Cycling conditions: 95°C 30 s; 40 cycles of 95°C 5 s, 60°C 30 s; followed by melt-curve analysis (95°C 15 s, 60°C 60 s, 95°C 15 s). m^6^A stoichiometry was calculated from the ΔCt between Set N and Set X and normalized against a synthetic RNA Oligo (m^6^A) standard curve (R² ≥ 0.99). All reactions were performed in ≥3 technical replicates.

### DRS data analysis

For Nanopore direct RNA sequencing (DRS) on R004 flow cells(PromethION R9.4.1 flow cell, Oxford Nanopore Technologies), raw electrical signals were processed using Dorado (Oxford Nanopore, v0.1; sup model) for high-accuracy, real-time base calling, with built-in demultiplexing enabled. Adapter and barcode sequences wee further identified and trimmed using Porechop_ABI (v0) under default settings. Cleaned reads were aligned to the human reference genome (hg38, GENCODE release) using minimap2 (v2) with splice-aware parameters (-ax splice -uf -k14) optimized for direct RNA data. Alignment output was stored in BAM format for downstream analyses.

For RNA modification analysis, aligned reads were processed with xPore (v2) to detect site-specific m^6^A modifications, while Nanopolish-polyA (v1.1) was applied to estimate poly(A) tail lengths from signal-level data. Run-level and per-sample quality control were assessed using NanoQC (v1) and NanoPlot (v1.2), which provide detailed summaries of read quality, length distributions, and yield statistics.

## Results

**Figure 1:**
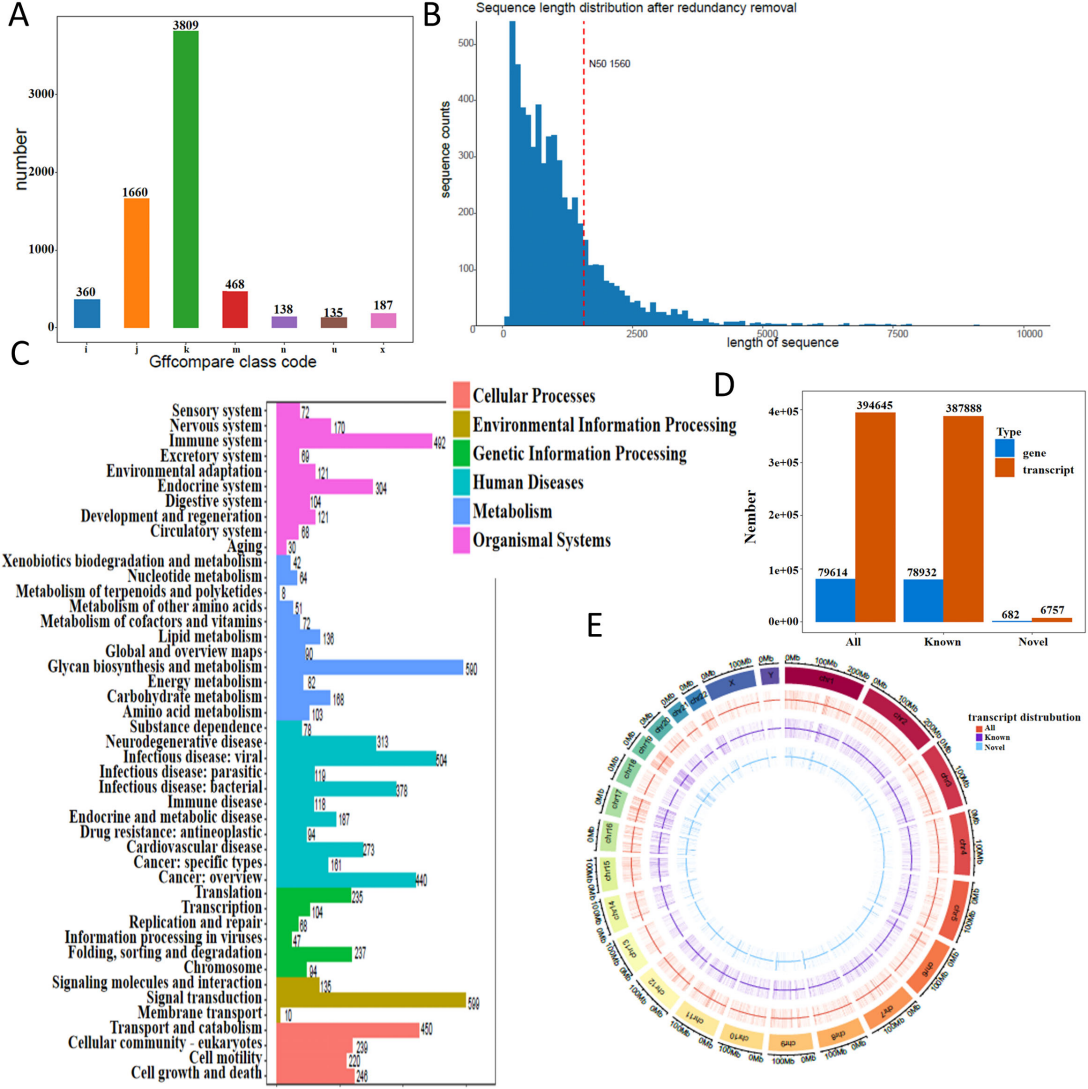
Transcriptome landscape revealed by direct RNA sequencing. **(A)** Distribution of novel transcript types identified in THP-1 macrophages. **(B)** Length distribution of coding sequences (CDS) from novel transcripts. The x-axis represents CDS length, and the y-axis indicates the number of transcripts within each length range. The red dashed line marks the N50 value. **(C)** KEGG pathway classification of annotated transcripts. **(D)** Summary of transcript and gene counts, categorized into all, known, and newly identified groups. **(E)** Transcript density map across the genome. Density was calculated in 500 kb windows and visualized using a circos plot.

Following low-quality read filtering, high-confidence data were retained for downstream analysis. We used *gffcompare* (v0.12.1; parameters: -R -C -K -M) to compare the resulting non-redundant transcript set with annotated reference transcripts, leading to the discovery of novel transcripts and genes that expand current genome annotations. In transcript classification, gffcompare assigns a class code to each assembled transcript relative to the reference annotation. Common codes include = (exact intron chain match), c (query fully contained within a reference transcript), j (sharing at least one splice junction but with a different overall structure), u (novel or intergenic, with no overlap to annotation), o (generic exonic overlap without a more specific match), p (possible polymerase run-on extending beyond the annotated 3′ end), i (entirely contained within a reference intron), x (exonic overlap on the opposite strand, i.e., antisense), s (within a reference intron but on the opposite strand), y (reference fully contained within the query), and e (single-exon overlap without full structural concordance). These codes provide a standardized framework for interpreting the relationship between novel assemblies and known reference transcripts. The distribution of novel transcript types is shown in **Figure 1A**. Coding sequence (CDS) prediction was performed for these novel transcripts, and the distribution of CDS lengths is shown in **Figure 1B**. Functional annotation using KEGG pathways categorized the transcripts into distinct biological pathways (**Figure 1C**). Transcript and gene counts were summarized for all, known, and newly identified categories (**Figure 1D**). We further analyzed the genomic distribution of transcripts on both strands in 500 kb windows and visualized their density using a *Circos* plot generated with the *circlize* R package (**Figure 1E**).

### Alternative splicing events induced by MTB infection in THP-1 cells

**Figure 2:**
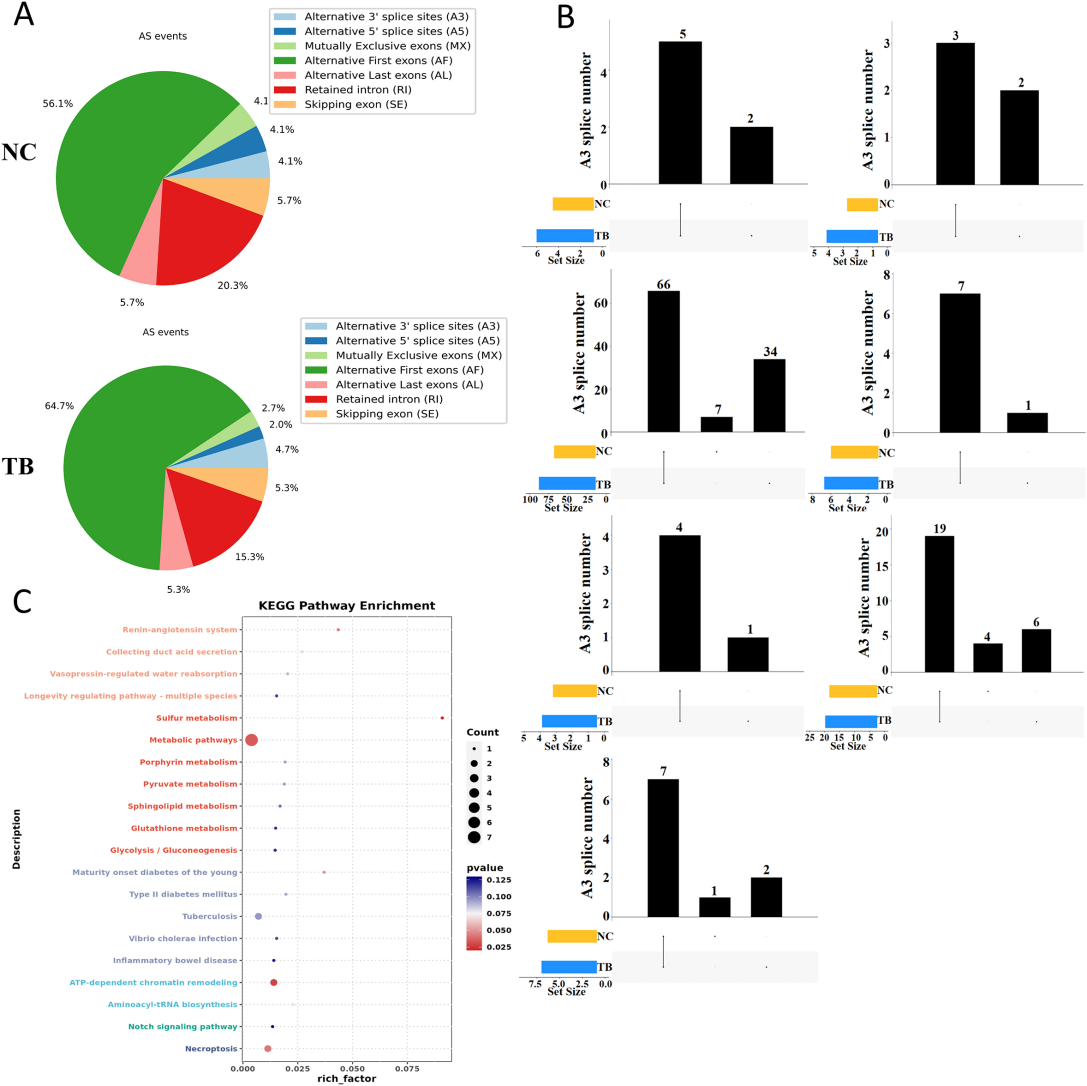
Alternative splicing dynamics following *Mycobacterium tuberculosis* infection. **(A)** Summary of alternative splicing events in THP-1 macrophages 48 hours post-infection. Event types include exon skipping (SE), mutually exclusive exons (MX), alternative 5′ splice site (A5), alternative 3′ splice site (A3), intron retention (RI), alternative first exon (AF), and alternative last exon (AL). **(B)** UpSet plot of alternative splicing events across groups. The lower left bar plot indicates the total number of events per group. The matrix layout and upper bar plot display the number of splicing events shared between or unique to each group. Events were considered valid only if consistently detected across biological replicates. **(C)** KEGG pathway enrichment analysis (dot plot) of transcripts with significantly altered splicing lengths.

We next examined the landscape of alternative splicing in control and MTB-infected THP-1 macrophages. Compared to the control group, MTB infection led to a notable increase in alternative splicing at the first exon and a decrease in intron retention events (**Figure 2A**). MTB infection also induced new splicing sites across all types of alternative splicing events (**Figure 2B**). Notably, events such as alternative first exon usage (AF), skipped exons (SE), and intron retention (RI) included both shared and novel splicing sites in the MTB-infected cells, with their overall numbers increasing. In contrast, alternative last exon (AL), alternative 5’ splice site (A5), alternative 3’ splice site (A3), and mutually exclusive exons (MX) showed entirely distinct splicing sites compared to controls, and their numbers decreased.

These findings suggest that MTB infection significantly rewires RNA splicing patterns. Further KEGG enrichment analysis of genes undergoing alternative splicing revealed that MTB infection specifically alters splicing in genes involved in metabolism, host-pathogen interactions, and inflammation-related pathways (**Figure 2C**).

### Poly(A) tail dynamics as a regulatory mechanism during MTB infection

**Figure 3:**
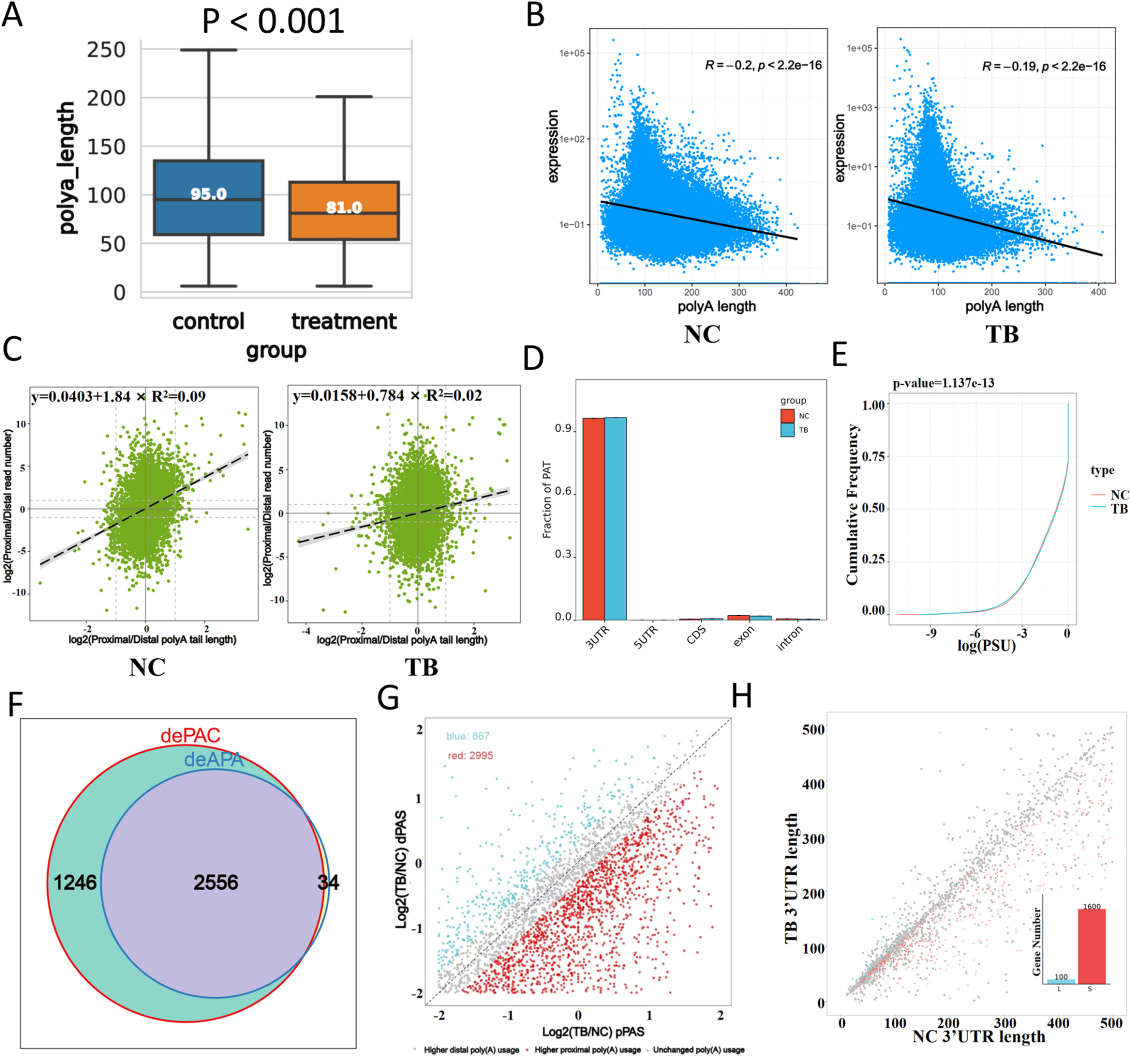
Polyadenylation changes in response to Mtb infection. **(A)** Distribution of poly(A) tail lengths 48 hours after infection. **(B)** Correlation between poly(A) tail length (x-axis) and transcript expression level (y-axis). **(C)** Relationship between proximal/distal site usage and poly(A) tail length. The x-axis indicates poly(A) tail length ratios, and the y-axis shows the abundance ratio of proximal to distal isoforms. **(D)** Distribution of polyadenylation sites (PATs) across gene regions. The x-axis represents gene structural elements, and the y-axis shows the proportion of PATs. **(E)** Global comparison of poly(A) site usage. The cumulative distribution function (CDF) plot compares the log-transformed usage values between groups. For example, at y = 0.25, a higher x-value in the treated group indicates greater site usage than the control. **(F)** Venn diagram showing overlap between differentially polyadenylated genes (dePAC: significant change in poly(A) activity; deAPA: significant change in site usage between groups). **(G)** Preferences for proximal versus distal poly(A) site usage. The x-axis represents the log ratio of proximal site usage; the y-axis represents the log ratio for distal site usage. Blue dots indicate genes preferring distal sites in the infected group; red dots indicate genes preferring proximal sites. **(H)** 3′UTR length variation analysis. The x-axis shows 3′UTR lengths in control samples, and the y-axis shows those in infected samples. ‘L’ and ‘S’ denote lengthening and shortening events, respectively. **(I)** KEGG pathway enrichment analysis (dot plot) of transcripts with significantly altered poly(A) tail lengths.

To explore the role of polyadenylation in MTB-infected macrophages, we analyzed poly(A) tail dynamics post-infection. MTB infection resulted in a global shortening of poly(A) tails, as reflected by a significant reduction in tail length (**Figure 3A**). Moreover, we observed a pronounced negative correlation between poly(A) tail length and gene expression, which became even stronger upon MTB infection. Genes with poly(A) tails around 100 nucleotides in length showed significantly increased expression (**Figure 3B**).

Correlation analysis between proximal/distal isoform abundance and poly(A) tail length revealed a weakened association in MTB-infected cells (**Figure 3C**). Polyadenylation site (PAT) mapping demonstrated increased usage within the 3’UTR and coding sequences (CDS), with a corresponding decrease in exonic and intronic regions (**Figure 3D**). Global comparison of poly(A) site usage patterns suggested functional relevance (**Figure 3E**). We identified genes with significantly altered poly(A) activity and site usage, performed Venn analysis to highlight those with the most dramatic shifts in polyadenylation patterns (**Figure 3F**).

Interestingly, MTB-infected cells preferentially used **distal** poly(A) sites, whereas control cells favored **proximal** sites (**Figure 3G**). 3’UTR length analysis further confirmed significant shifts (**Figure 3H**). KEGG pathway clustering of genes with altered poly(A) tail lengths showed enrichment in pathways related to infection and inflammation, highlighting polyadenylation as a key regulatory layer in the host response to MTB.

### m6A RNA methylation plays a key role in MTB-infected macrophages

**Figure 4:**
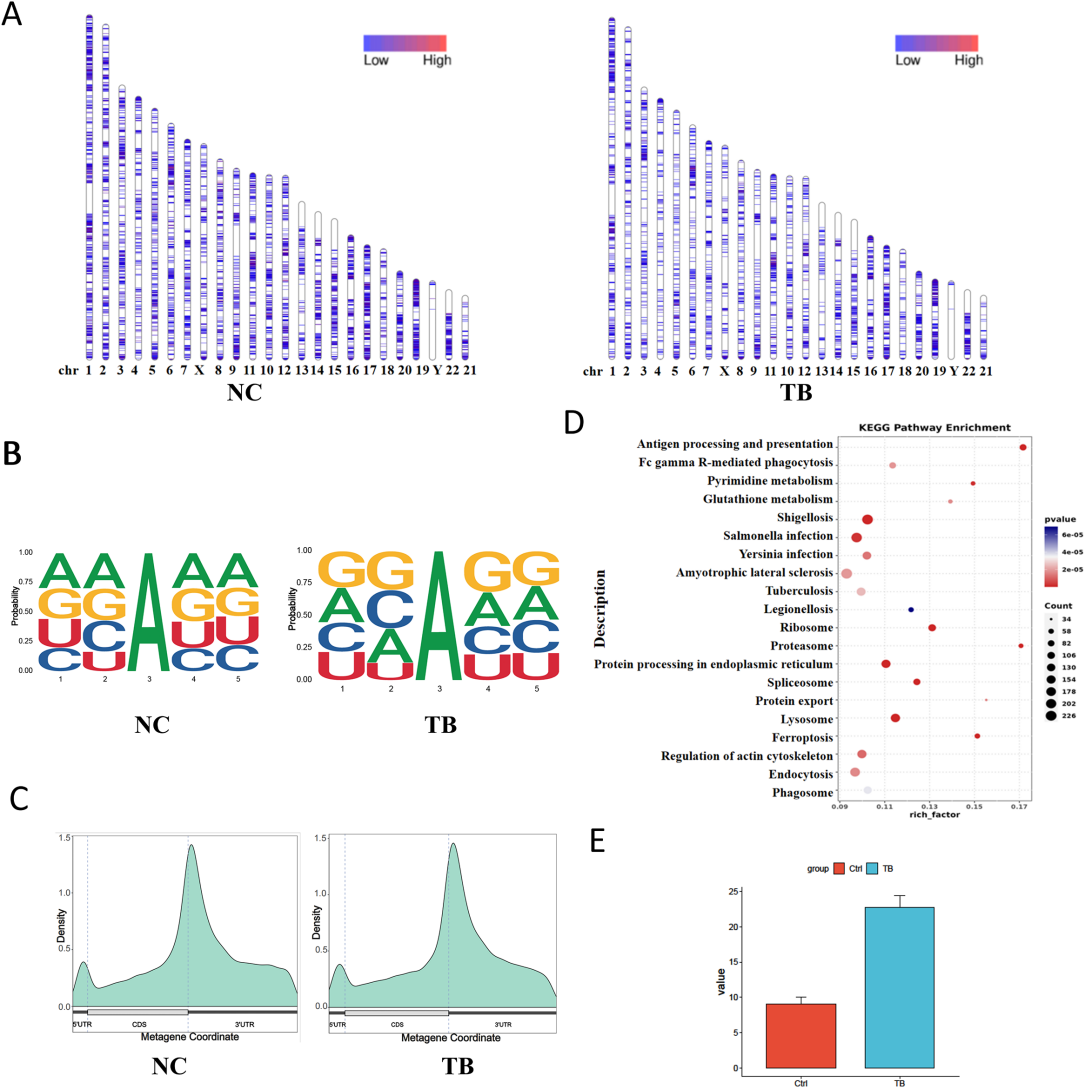
m6A RNA methylation landscape and functional impact. **(A)** Chromosomal distribution of m6A peaks. Color intensity represents the density of m6A methylation sites. **(B)** Sequence motif surrounding m6A sites (± 2 nucleotides). The x-axis shows base positions, with total bar height indicating conservation and individual base heights representing relative frequency. **(C)** Distribution of m6A sites across gene structural elements. The x-axis indicates gene features; the y-axis shows m6A density. **(D)** KEGG pathway enrichment analysis (dot plot) of transcripts with differential m6A modification. **(E)** m6A levels on the *VEGFA* gene detected via SELECT assay.

We first assessed the genome-wide distribution of m6A methylation sites in THP-1 macrophages upon *Mycobacterium tuberculosis* (MTB) infection. A marked reduction in m6A levels was observed on several chromosomes, including chromosomes 3, 5, and 14 (**Figure 4A**). To characterize sequence specificity, we extracted 2 nucleotides upstream and downstream of each m6A site to form 5-mer motifs. MTB infection altered both the nucleotide composition and frequency of these motifs (**Figure 4B**).

Annotation of m6A sites relative to transcript features revealed decreased methylation in the 5′UTR and coding sequence (CDS) regions, and an increase in the 3′UTR following infection (**Figure 4C**). KEGG pathway enrichment analysis of transcripts with differential m6A modifications indicated significant remodeling of MTB-related genes. These differentially modified transcripts were enriched in pathways related to host-pathogen interaction and cellular metabolism (**Figure 4D**). We also validated m6A levels on the VEGFA gene via SELECT assay (**Figure 4E**).

### Role of pseudouridine (Ψ) modification in the context of TB infection

**Figure 5:**
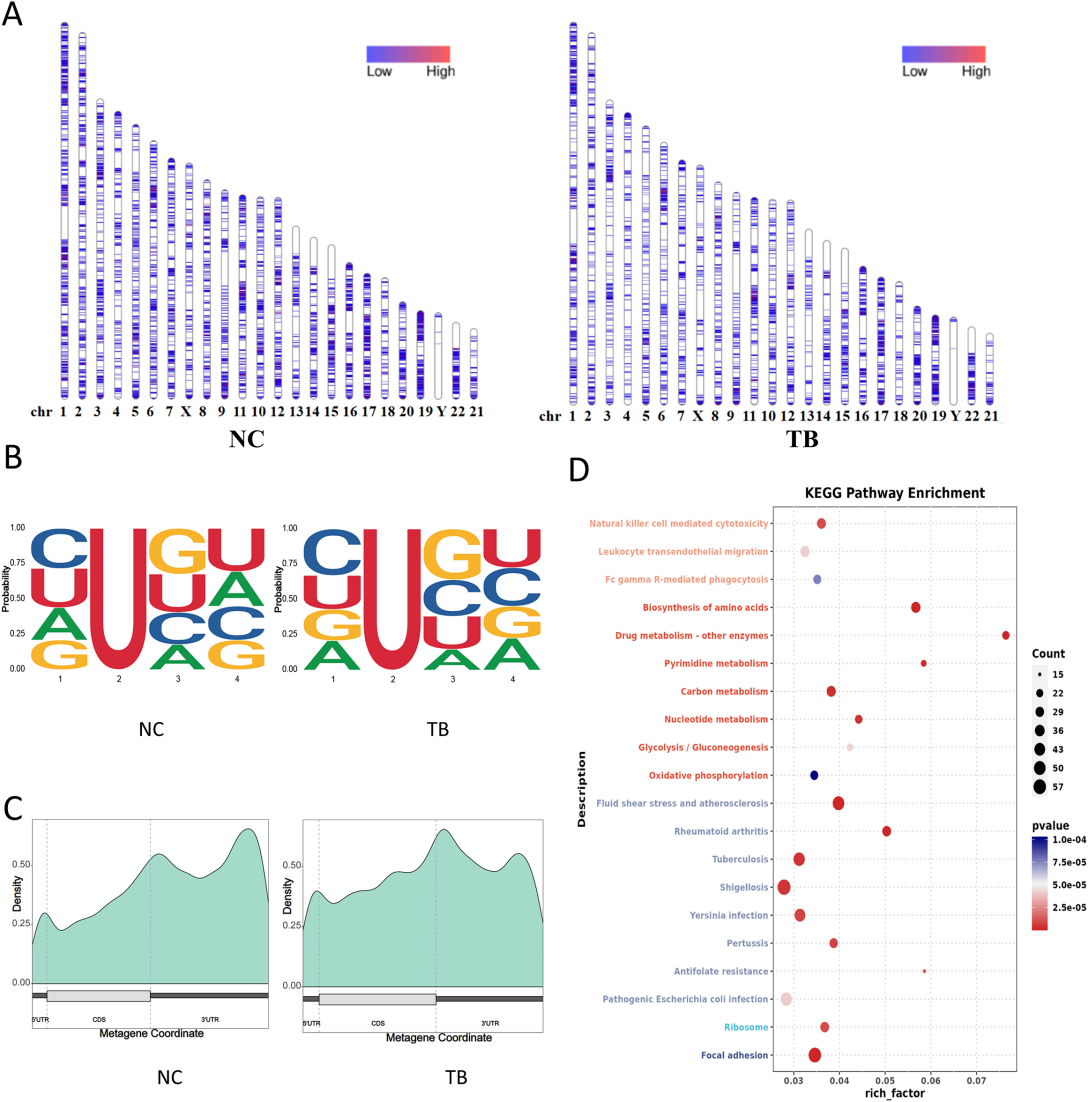
Pseudouridine (Ψ) modification landscape. **(A)** Chromosomal distribution of Ψ sites. Color intensity denotes Ψ site density. **(B)** Sequence motif surrounding Ψ sites (−1 to +2 bp). See **Figure 4B** for interpretation. **(C)** Distribution of Ψ sites across transcript structural regions. **(D)** KEGG pathway enrichment of transcripts with differential Ψ modification (dot plot).

We next examined pseudouridine (Ψ or PSU) modifications. MTB infection significantly reduced genome-wide PSU abundance, with chromosome 12 showing a notable decline (**Figure 5A**). We generated 4-mer motifs by extending 1 nucleotide upstream and 2 downstream of each Ψ site. The base composition and frequency of Ψ motifs were altered upon infection (**Figure 5B**).

Region-specific analysis indicated a significant increase in Ψ modifications within CDS regions, alongside a reduction in the 3′UTR (**Figure 5C**). KEGG enrichment analysis of transcripts with altered Ψ modifications highlighted changes in MTB-related genes, especially those involved in metabolic and infection-associated pathways (**Figure 5D**).

Moreover, the genome-wide distribution of 5-methylcytosine (m5C) sites was also affected by MTB infection, with an overall reduction across the macrophage transcriptome (**Supplementary Figure S1A**). For motif analysis, we extracted 2 nucleotides upstream and downstream of each site to generate 5-mers. MTB infection led to significant changes in both the base composition and frequency of m5C motifs (**Supplementary Figure S1B**).

Region-specific mapping showed that m5C levels increased significantly in the CDS and 5′UTR regions, while they decreased in the 3′UTR (**Supplementary Figure S1C**). KEGG clustering of differentially modified transcripts revealed that m5C modifications upon MTB infection were associated not only with metabolic pathways, but also prominently with inflammation-related genes and RNA-binding protein pathways (**Supplementary Figure S1D**).

Lastly, we investigated inosine (I) modifications. MTB infection caused a global reduction in inosine editing, particularly on chromosome 1 (**Supplementary Figure S2A**). To identify characteristic sequence motifs, we extended 2 bases both upstream and downstream of each inosine site to create 5-mers. The nucleotide composition and frequency of inosine motifs were substantially altered in infected cells (**Supplementary Figure S2B**).

Mapping the distribution of inosine sites across transcript regions revealed increased editing in the 5′UTR and CDS regions, and a corresponding reduction in the 3′UTR (**Supplementary Figure S2C**). KEGG enrichment analysis of transcripts with altered inosine modifications indicated enrichment in metabolic, apoptosis, and RNA/protein interaction pathways, underscoring the functional consequences of RNA editing changes during infection (**Supplementary Figure S2D**).

## Discussion

Our study provides the first high-resolution, third-generation sequencing-based epitranscriptomic map of human macrophage responses to *Mycobacterium tuberculosis*. Using ONT DRS, we demonstrate that Mtb infection leads to extensive changes across multiple RNA processing layers, including alternative splicing, APA, and RNA modifications.

The observed increase in exon skipping and first exon alternative usage suggests dynamic transcriptome remodeling aimed at fine-tuning host immune responses. Notably, several key immune-regulatory genes underwent isoform switches, aligning with previous findings on transcriptome plasticity during bacterial infections (Cun et al., 2023; Chu et al., 2024).

We found that poly(A) tail lengths were globally shortened upon infection, consistent with translational repression observed during cellular stress or immune activation. Genes with 100 bp polyA tails were disproportionately upregulated, highlighting a possible length-optimal window for transcript stability in infected macrophages.

Our analysis also reveals distinct patterns of m6A, m5C, Ψ, and inosine modifications in response to infection. Reduced m6A in 5′UTRs and CDS regions may impact cap-independent translation, while increased 3′UTR methylation could affect mRNA decay or microRNA targeting. The altered distribution of pseudouridine and inosine in CDS and 5′UTRs further suggests translational recoding and immune sensor evasion mechanisms.

Importantly, pathway enrichment analysis demonstrated that genes exhibiting these epitranscriptomic changes are significantly involved in inflammation, apoptosis, metabolic pathways, and host immune regulation—mirroring the known pathophysiology of Mtb infection.

In conclusion, our findings establish third-generation DRS as a powerful approach to unravel complex RNA regulatory networks in infectious disease contexts. Future studies integrating single-cell DRS and ribosome profiling could further delineate how RNA modifications influence translation during Mtb infection and latency.

While our study provides a comprehensive view of the transcriptional and epitranscriptomic landscape in macrophages upon *Mycobacterium tuberculosis* (Mtb) infection using direct RNA sequencing (DRS), several limitations should be noted. First, the analysis was conducted in THP-1-derived macrophages, which, although widely used, may not fully capture the complexity and heterogeneity of primary human macrophages or tissue-resident immune cells *in vivo*. Second, while we identified associations between epitranscriptomic changes and functional pathways, further mechanistic studies are required to validate the causal roles of specific RNA modifications in modulating host immune responses during infection. Future work incorporating primary cells, larger cohorts, and functional perturbation assays will be essential to address these gaps and fully elucidate the regulatory architecture underlying host–Mtb interactions.

In contrast to previous studies that have primarily characterized host transcriptional responses to *Mycobacterium tuberculosis* infection using short-read sequencing, our work leverages Oxford Nanopore direct RNA sequencing to capture full-length transcripts and simultaneous epitranscriptomic modifications. While earlier reports have highlighted limited RNA modification changes or alternative splicing events, we provide a comprehensive map of m6A, m5C, pseudouridine, and inosine dynamics, along with global poly(A) tail shortening and alternative polyadenylation shifts. This allows us to delineate regulatory layers that were previously unappreciated, linking transcript isoform diversity and RNA modifications to functional pathways involved in inflammation, metabolism, and apoptosis. Our study therefore extends the current literature by not only confirming known transcriptional responses but also uncovering novel post-transcriptional and epitranscriptomic mechanisms that may influence host-pathogen interactions during Mtb infection.

Despite the depth of our DRS survey, the study remains descriptive and lacks direct functional validation. We show strong correlative evidence that Mtb infection redistributes m^6^A, m^5^C, Ψ and inosine marks and alters poly(A) site choice, but we do not demonstrate that these changes are causally required for bacterial survival or for the host response. CRISPR-based epitranscriptomic editors (dCas13-METTL3, dCas13-ALKBH5, etc.) or metabolic labelling followed by miCLIP were not employed to confirm the exact modified residues, nor were infections performed with Mtb mutants lacking ESX-1 or other RNA-binding effectors to test whether the observed shifts are microbe-driven. Additionally, all experiments were conducted in the THP-1 cell line, which may not recapitulate primary macrophage heterogeneity or *in-vivo* hypoxic granuloma environments. Future work should integrate epitranscriptome perturbation, orthogonal modification mapping and infections of PBMC-derived or BMDM macrophages to establish the mechanistic necessity of the identified RNA modifications in Mtb persistence and immune evasion.

## Data Availability

The original contributions presented in the study are publicly available. This data can be found here: https://ngdc.cncb.ac.cn/omix/release/OMIX009081.

## Supplementary material

Supplementary Figure 1 m5C RNA methylation profile. (**A)** Chromosomal distribution of m5C sites. **(B)** 5-mer sequence motifs centered on m5C sites. **(C)** m5C site density across gene features. **(D)** KEGG enrichment analysis of transcripts with altered m5C modification.

Supplementary Figure 2 Inosine RNA editing dynamics. **(A)** Chromosomal distribution of inosine-modified sites. **(B)** 5-mer motif analysis surrounding inosine sites. **(C)** Distribution of inosine sites across gene structural elements. **(D)** KEGG pathway enrichment analysis of transcripts with differential inosine modification.
