# Supplementary figures and images for: Direct RNA sequencing reveals multilayered epitranscriptomic remodeling in macrophages upon Mtb infection
Source: Front Cell Infect Microbiol. 2025 Nov 11;15:1689553. doi: 10.3389/fcimb.2025.1689553 (PMC12644008; doi:10.3389/fcimb.2025.1689553)

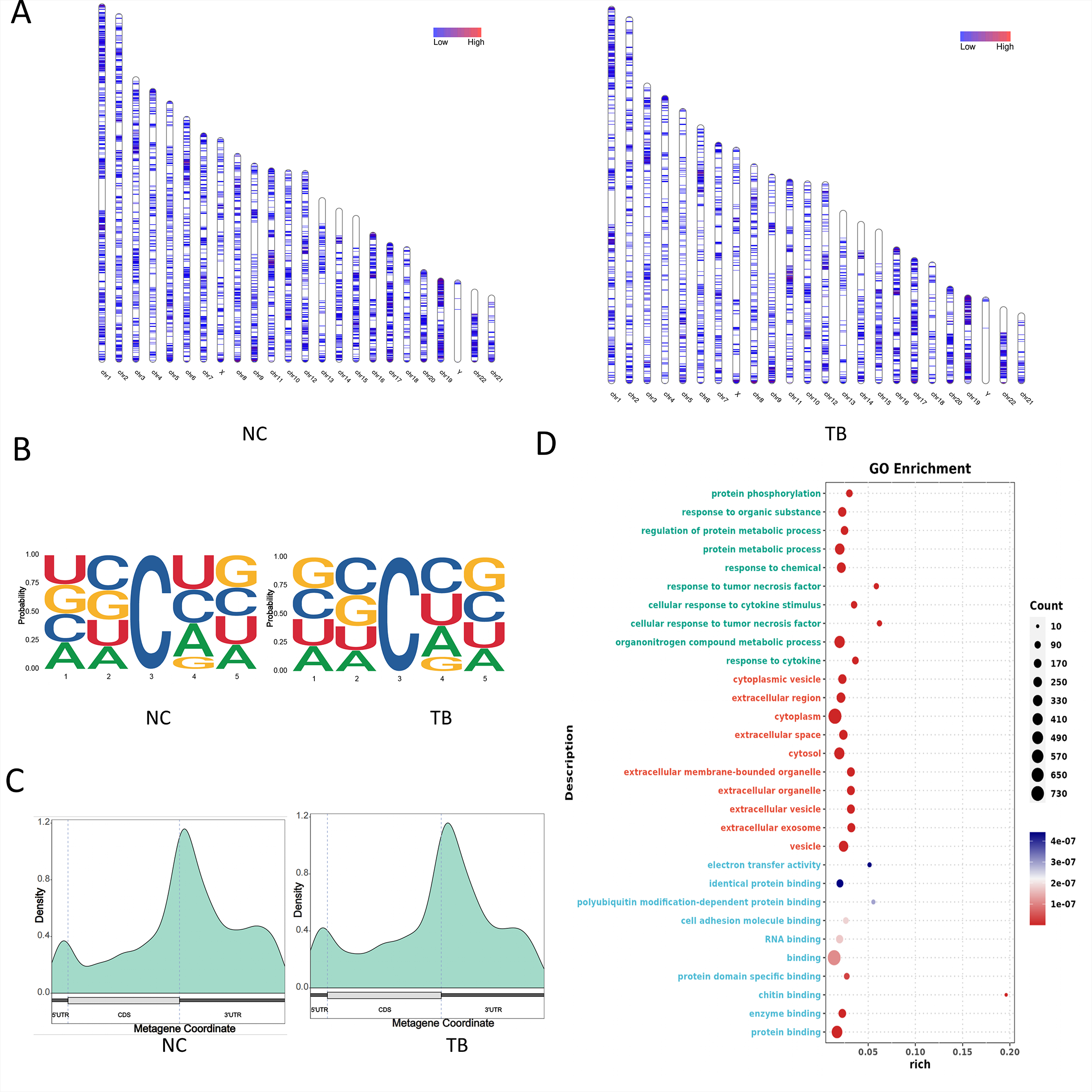

Supplement: Supplementary Figure 1 — m5C RNA methylation profile. (A) Chromosomal distribution of m5C sites. (B) 5-mer sequence motifs centered on m5C sites. (C) m5C site density across gene features. (D) KEGG enrichment analysis of transcripts with altered m5C modification. [file Image1.tif]

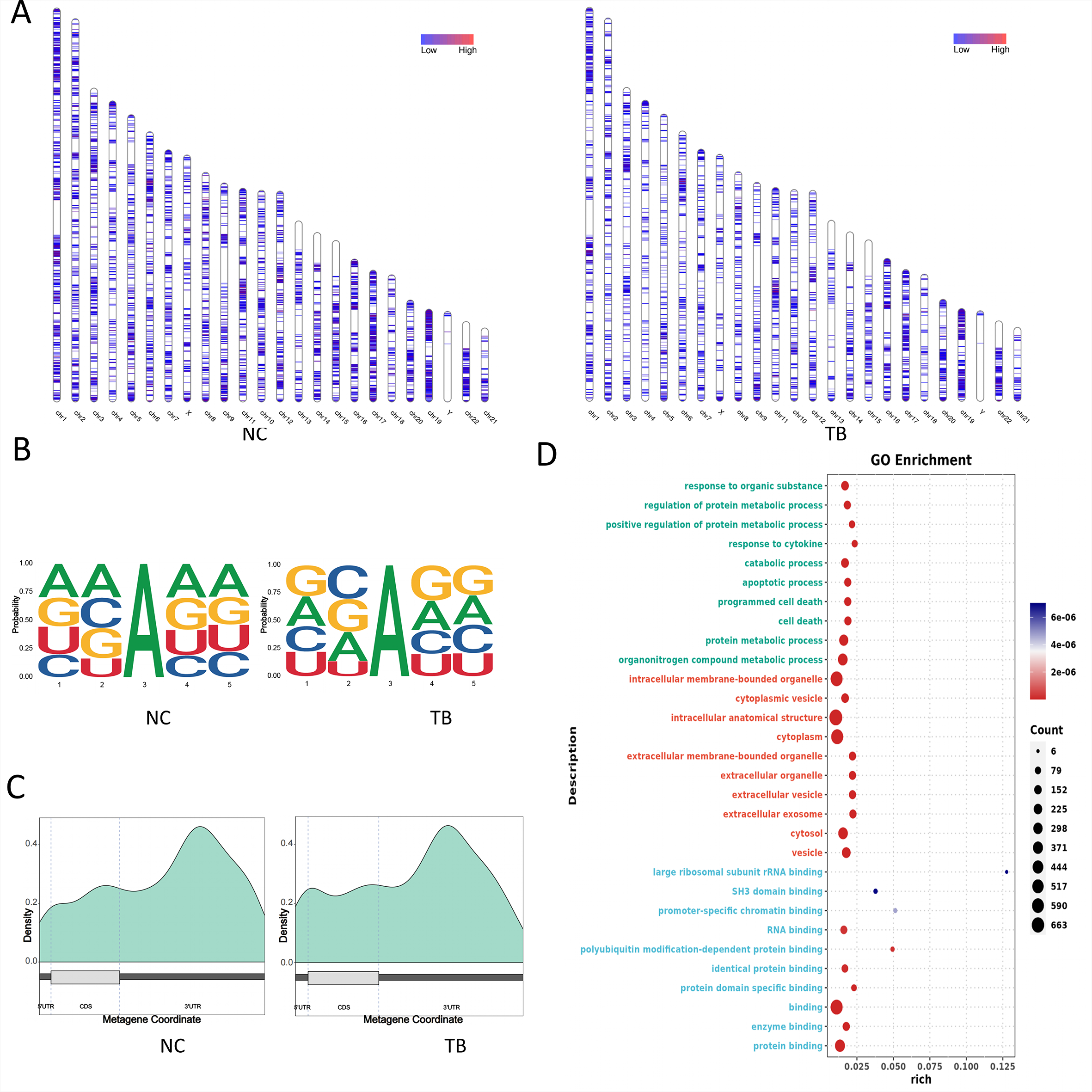

Supplement: Supplementary Figure 2 — Inosine RNA editing dynamics. (A) Chromosomal distribution of inosine-modified sites. (B) 5-mer motif analysis surrounding inosine sites. (C) Distribution of inosine sites across gene structural elements. (D) KEGG pathway enrichment analysis of transcripts with differential inosine modification. [file Image2.tif]
